# Effect of Classical Music on Depth of Sedation and Induction Propofol Requirements in Dogs

**DOI:** 10.3390/vetsci10070433

**Published:** 2023-07-03

**Authors:** Stefanos G. Georgiou, Aikaterini I. Sideri, Tilemachos L. Anagnostou, Pagona G. Gouletsou, Vassiliki G. Tsioli, Apostolos D. Galatos

**Affiliations:** 1 Clinic of Surgery, Faculty of Veterinary Science, School of Health Sciences, University of Thessaly, 43100 Karditsa, Greece; stegeorgiou@vet.uth.gr (S.G.G.); ksideri@vet.uth.gr (A.I.S.); vtsioli@vet.uth.gr (V.G.T.); 2 Anaesthesia and Intensive Care Unit, School of Veterinary Medicine, Faculty of Health Sciences, Aristotle University of Thessaloniki, 54627 Thessaloniki, Greece; tanagnos@vet.auth.gr; 3 Clinic of Obstetrics and Reproduction, Faculty of Veterinary Science, School of Health Sciences, University of Thessaly, 43100 Karditsa, Greece; ngoule@vet.uth.gr

**Keywords:** acepromazine, butorphanol, dog, music, propofol, sedation

## Abstract

**Simple Summary:**

Music therapy seems to exhibit beneficial properties for a variety of human medical conditions, with some benefits being reported even throughout the perioperative period. In accordance with the human literature, the responses of animals’ exposure to musical auditory stimuli as a means of environmental enrichment seem to be encouraging, but very little is known about the effect of music when used as an adjuvant to anaesthetics. The aim of this study was to evaluate the effect of classical music on the depth of sedation and propofol requirements for the induction of anaesthesia in 20 healthy dogs. Each dog was sedated three times, using a mild premedication protocol, and was exposed to Chopin, Mozart and no-music treatments for 90 min via loudspeakers, with a 3-month gap between each one of the three auditory interventions. The dogs’ exposure to classical music resulted in higher sedation scores and approximately 20% lower propofol dose requirements for the intubation of the trachea. This study proposes that music may prove a simple, safe and effective nonpharmacologic adjunct when used as part of a mild premedication protocol.

**Abstract:**

The main objective of this prospective, randomized, blind, cross-over experimental study was to evaluate the effect of classical music on the depth of sedation and propofol requirements for the induction of anaesthesia in dogs. Twenty dogs were involved, and each was subjected to three different treatments with a 3-month gap: Chopin music, Mozart music, and no music, via loudspeakers. The dogs were premedicated with acepromazine and butorphanol by intramuscular injection, and anaesthesia was induced using propofol intravenously. To compare the depth of sedation and propofol requirements for the induction of anaesthesia among the different treatments, we utilized non-parametric tests (Kruskal–Wallis test) for the depth of sedation due to a slight deviation from the normal distribution and parametric (ANOVA) for propofol requirements. When exposed to music (Chopin or Mozart), dogs exhibited deeper sedation and required less propofol for their intubation compared to the no-music treatment (*p* < 0.05). Exposure to classical music had a positive impact on the level of sedation, and more profound central nervous system depression seemed to contribute to approximately 20% lower propofol dose requirements for tracheal intubation. Therefore, classical music during the preoperative period appeared to exert a beneficial effect, at least when applying the specific pre-anaesthetic medications used in the present study.

## 1. Introduction

Music therapy seems to have been popular since antiquity, when, as legend has it, Pythagoras of Samos used musical sounds for treating patients. Recently, music therapy seems to be strongly supported by scientific evidence and provided by a number of studies examining the neurochemical changes that occur due to music exposure, in different fields of medicine, such as psychiatry, intensive care units (ICU) and during the perioperative period, due to its many beneficial effects [1,2,3,4,5,6,7,8]. Music therapy seems to have a positive impact on the management of symptoms such as pain, fatigue, and anxiety, resulting in an improvement in the quality of life of palliative care patients, and more specifically, Mozart’s music contributes to an acute decrease in epileptic activity and seizure control, as add-on therapy, in children and adults with epilepsy or even status epilepticus [7,9,10,11]. In surgical patients, advantages such as stress reduction, low cost, ease, and safety, could render music an attractive complementary tool during a balanced anaesthetic approach. Patients during the perioperative period often report high anxiety levels, which may potentiate the perception of pain, not to mention the severity of the pain related to surgery. Furthermore, surgical trauma induces physiologic stress and dysfunction of the autonomic nervous system (ANS) [8]. Therefore, physicians can try to reduce the magnitude of these problems by administering sedatives and analgesics, which, however, may have a number of detrimental side effects or can complicate and prolong patient recovery [5,8,12].

In relation to ICU patients, it has been reported that approximately 70–80%, especially those being mechanically ventilated, demonstrate high anxiety levels. If not regulated, anxiety stimulates the sympathetic nervous system (SNS), increasing the work of breathing and fatigue and substantially delaying ventilator weaning. SNS arousal can elicit a variety of adverse effects, such as vasoconstriction, myocardial stimulation and bronchoconstriction [12]. The latter predisposes patients to elevate airway resistance, increases the work of breathing and oxygen demand, as well as muscle and generalized fatigue. Furthermore, SNS stimulation can also result in an elevated rate and depth of respiration, as well as an elevated heart rate. The attenuation of these symptoms involves the administration of sedative and analgesic medications, and these agents, especially if administered at high doses and for prolonged periods, can predispose to hypotension, gut dysmotility, immobility or delirium, which can lead to prolonged ventilation and increased ICU stays, while continuous sedation and prolonged ICU stays seem to be responsible for increased rates of organ failure and the reintubation of the trachea. In the context of ICU aesthetic agent reduction, nonpharmacological approaches such as music therapy can diminish stress responses and decrease anxiety during mechanical ventilation. This can result in a lower cardiac workload and oxygen consumption and consequently promote more effective ventilation and accelerate ventilator weaning [12].

A variety of explanations have been proposed about the way music exhibits its effect. Brain imaging studies in humans have illustrated that music exposure induces effects in both cortical and subcortical areas involving memory and motor functions, as well as areas relating to emotions, such as the limbic and paralimbic regions [12]. A variety of studies have described a significant decrease in the respiratory rate, heart rate, and arterial pressure in music-exposed ventilator-assisted patients, and while the underlying psychobiological mechanisms still remain unknown, a hypothesis of how these music-induced modifications occur is that they might be due to the modulation of the hypothalamic–pituitary–adrenal axis [12]. Therefore, music seems to reduce clinical morbidity by decreasing sympathetic activation and preserving the metabolic and immune homeostasis of stress metabolism and immune functions via a strengthening parasympathetic cardiac cholinergic output from the myelinated neurons in the nucleus ambiguous of the vagus nerve [8]. Furthermore, perioperative music exposure seems to contribute to reduced stress levels [3], reduced cortisol and catecholamine values [4,6,8,12], and the production of endogenous opioids and oxytocin [8], while the music-sparing effect of anaesthetics has been demonstrated [1,2,4,6]. Up to now, studies on domestic animals have mainly focused on the effects of music on welfare and stress responses and not on its effect on sedation and anaesthesia. Music, especially classical music, seems to exert a beneficial effect as an enrichment method in animal species such as bovines [13], pigs [13,14] and rats [15,16,17]. In dogs, classical music appears to reduce environmental stress both in kennelled [18,19,20,21,22,23,24] and owned dogs during a visit to the veterinary hospital [25]. However, the authors are aware of only four studies that aimed to evaluate the impact of music in domestic animals when anaesthetic agents were administered in dogs under sedation [26], in cats [27,28] and in dogs under general anaesthesia [29].

In humans, self-selected music seems to be a cornerstone of the music application, taking into consideration that familiar music can induce emotional responses and have a positive effect on physiological responses [8,12]. However, it goes without saying that self-selected music cannot be applied to domestic animals. It has been suggested that the music genre, rhythm and sound level may affect the impact of music on animal species. Classical music seems to evoke desirable responses in cats under general anaesthesia [27,28] and dogs [18,19,21,23,24]. Dogs seem to prefer slow rhythm music [20,22,23,24,25], just like humans, to whom slow tempos between 60 and 80 beats per minute elicit beneficial responses [8,12]. Finally, the volume of musical stimulus is always set by the patient in human medicine [1,2], and sound volume levels of 60–70 dB have been proposed [6,30]. No studies investigating the proper volume of acoustic stimulus in dogs or other domestic animals have been conducted. However, acoustic stimuli >80 dB seem to have a negative impact on bovine [13], pigs [31], rats [32,33] and hens [34], while, in dogs, when sedated with dexmedetomidine, the quality of sedation was negatively affected by an acoustic stimulus (noise conditions) of 80–85 dB [26]. On the other hand, musical stimuli of 43–73 dB in non-sedated dogs [20,21,23,24,25,35] and classical music of <80 dB in cats under general anaesthesia [27,28] have shown a beneficial effect.

The objective of the present study was to investigate whether classical music has a potentially beneficial effect on the depth of sedation and propofol requirements for the induction of anaesthesia in dogs. We hypothesized that classical music could enhance the central nervous system (CNS) depression induced by specific pre-anaesthetic medications and subsequently lower the propofol requirements for the induction of anaesthesia in dogs.

## 2. Materials and Methods

### 2.1. Animals

This was a prospective, randomized, blind, cross-over, experimental study. The experimental protocol was approved by the Animal Ethics Committee of Greece (license number 1117/45296 21 March 2017), which confirmed that it complied with the standards of the national and EU legislation (Directive 2010/63/EU for animal experiments) regarding animal experimentation.

Twenty healthy (American Society of Anaesthesiologists status I), adult purpose-bred laboratory Beagles (7 females and 13 males), who were scheduled to undergo skin surgery (for another study protocol), were used in this study. Their mean ± SD age and weight were 3.6 ± 1.3 years and 11.9 ± 2.2 kg, respectively. Their physical examination, auditory function, complete blood count and serum biochemical analyses were within normal limits before the commencement of the experimental protocol. Their auditory function was assessed by an otoscopic examination of the external ear and tympanum radiography of the tympanic bullae for a neurologic examination as well as clinical observations for a response to a sound stimulus outside the animal’s visual field. An 8 h fasting period and 2 h of deprivation of water were implemented before the initiation of the experimental procedure.

### 2.2. Study Design

Using an online randomization tool (Research Randomizer, http://www.randomizer.org, accessed on 15 April 2017), we created a dataset indicating the music treatment that the dog would follow during every intervention. We asked for 20 sets of three random numbers per set, with every number indicating the different music treatments. A strict 3-month interval between the consecutive interventions in the same dog was considered adequate to minimize bias. A special effort was made to minimize the animals’ stress before and during the experimental procedure, as it would probably interfere with the effect of the music on the depth of sedation. All dogs were unfamiliar with music listening as part of their daily routine. None of them had received any sedatives during the previous two months. Three treatment groups were formed according to the different types of musical stimulus applied for 90 min, and each consisted of the same 20 dogs. At first, the dogs listened to Chopin’s Music (Nocturne in D-flat major, Op. 27, No 2-lento sostenuto) (CM); in the second, they were exposed to Mozart Music (Sonata for two pianos in D-major, K.448, andante) (MM) while the third treatment was a No-Music control treatment (NM), in which the whole procedure was performed without listening to music. Music was provided via loudspeakers. The volume of the stimulus never exceeded 65 dB, as confirmed by the use of a decibel meter, and remained stable during the whole procedure (50–65 dB) while the playback of the musical stimulus was continuous.

### 2.3. Procedure

Music exposure during the preoperative period lasted 90 min in total, while during the NM treatment, the dogs remained in the same silent environment for the same time period. Forty-five minutes after music initiation, the dogs were premedicated with acepromazine (Acepromazine, Alfasan, The Netherlands) at 0.03 mg/kg and butorphanol (Dolorex, MSD, The Netherlands) at 0.1 mg/kg intramuscularly (IM). After another 45 min, the music was turned off, and the level of sedation was assessed by a blind treatment assessor. The cephalic vein was catheterized, and Lactated Ringer’s solution was administered at a rate of 5 mL/kg/h. Oxygen at 4 L/min flow-by was supplied for 10 min before and during the induction of anaesthesia with propofol (Propofol, Fresenius Kabi, Athens, Greece) with increments of 1 mg/kg up to 3 mg/kg intravenously (IV) and 0.5 mg/kg, thereafter, every 60 s, until the fulfilment of the predefined terms of intubation, as proposed by Gurney et al. [36]. Following every propofol bolus, the specified criteria for intubation were evaluated, consisting of a loss of jaw tone, the absence of resistance to the protraction of the tongue and the absence of gag reflex [36]. Propofol was administered by hand, and the total dose required for the intubation of the trachea was recorded. As already stated, the person assessing the depth of sedation and performing the induction of anaesthesia and tracheal intubation was blind to the type of treatment, i.e., the type of auditory stimulus.

### 2.4. Assessment of Sedation (Scoring System)

Sedation was blindly assessed using a composite descriptive scale (Table 1), which was adapted from another study [37], giving a score of 0 to 15 (0 = no sedation, 15 = well sedated) [36]. Sedation was assessed just before the dogs’ exposure to music and 90 min later, before the induction of anaesthesia. During that period, the dogs were left undisturbed in private cages, being exposed to the particular music treatment they were included in, or in total silence for the no music treatment. The sedation scores were recorded for each dog in each intervention.

### 2.5. Statistical Analysis

For the descriptive analysis of nominal and ordinal variables, we calculated the frequency and relative frequency. For scale variables (e.g., propofol requirements), we used the mean and standard deviation (SD) for these variables following their normal distribution [mean ± SD], while for those rejecting the hypothesis of normality (e.g., depth of sedation score) we utilized the median and interquartile range (IQR) [median (IQR)]. For the representation of the results, we applied means and boxplots accordingly.

To examine differences among the treatments, we employed ANOVA and a t-test for variables following the normal distribution and Kruskal–Wallis with Mann–Whitney for those violating normality. For all pairwise comparisons, post hoc analysis was performed by applying the Bonferroni correction to minimize the type I error. Statistical significance was defined as *p* < 0.05.

## 3. Results

### 3.1. Assessment of Sedation

The sedation scores just before the initiation of the experiment (i.e., exposure or no exposure to music) were zero, as all dogs were alert and fully conscious. At the time point of interest (90 min later), significantly higher sedation scores were detected in dogs in the CM 5.0 (2.5) and MM 6.0 (4.0) treatments compared to dogs in the NM treatment 2.0 (1.5) (Table 2).

**Figure 1:**
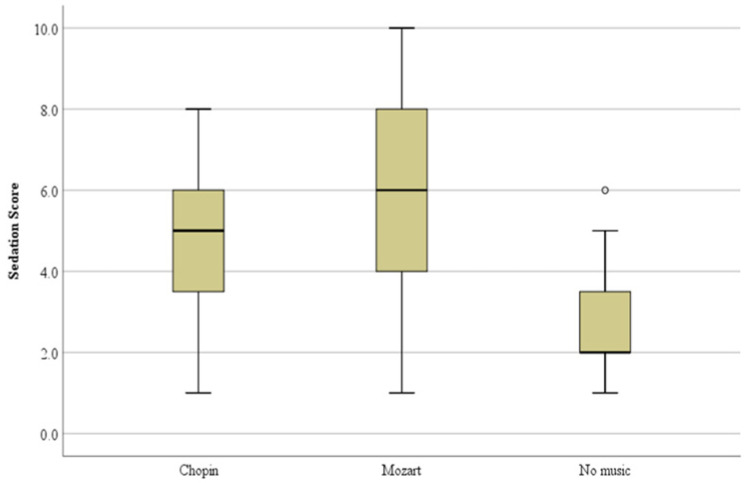
Boxplot of the sedation score among the 3 treatments (CM, MM and NM) 90 min after auditory stimulation and 45 min after premedication. The horizontal line inside the boxes denotes the median value, while the upper and lower whisker bounds denote the maximum and minimum values. The differences between the CM–NM and the MM–NM treatments were statistically significant (*p* < 0.01).

Based on the results of the above table, we can conclude that the sedation score among the three treatments was different (K-W 20.031, *p* < 0.01). During the post hoc comparisons of the sedation score, NM was different both from the CM (*p* < 0.01) and the MM (*p* < 0.01) treatments. These results are presented in the following boxplot (Figure 1).

### 3.2. Propofol Dose for Tracheal Intubation (mg/kg)

The propofol doses required for the intubation of the trachea were significantly lower in the CM (4.7 ± 0.9 mg/kg) and the MM (4.2 ± 0.8 mg/kg) treatments compared to the NM treatment (5.7 ± 0.9 mg/kg) (Table 3). Exposure to Chopin’s music seemed to reduce the propofol requirements for tracheal intubation by approximately 18%, while for Mozart’s music, the respective reduction was approximately 26%.

**Figure 2:**
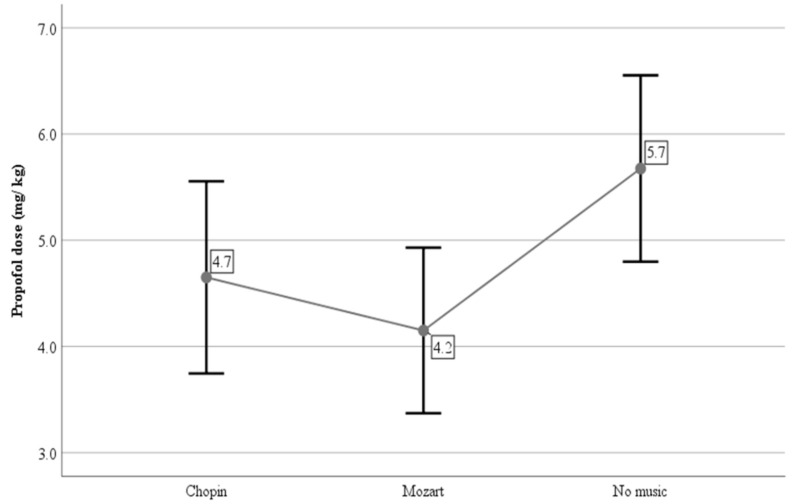
Means plot with ± SD whisker of propofol dose (mg/kg) for tracheal intubation in dogs among the 3 treatments (CM, MM, NM). The differences between the CM–NM and the MM–NM treatments were statistically significant (*p* < 0.01).

Based on the results of the above table, we can conclude that there was a statistically significant difference in the propofol values among the three treatments (F = 16.508, *p* < 0.05). To examine the treatments pair-wisely, we performed post hoc comparisons using the Bonferroni method to minimize the type I error. From the post hoc comparisons, we could conclude that the mean propofol value of the NM treatment was significantly higher than the CM and the MM treatments (*p* < 0.01 in both comparisons). These results are presented in the following means plot (Figure 2).

## 4. Discussion

The present study investigated the potential effects of music on a specific pre-anaesthetic protocol in dogs, considering the encouraging results of exposure to music in humans and domestic animals. Recently, two studies on cats [27,28] and two studies on dogs [26,29] reported the interaction between music and anaesthetic agents. In human medicine, the positive effect of listening to music, noted by most authors during the perioperative period, is primarily based on the selection of music by the patient [1,2,3,30].

The choice of the appropriate musical stimuli for this study was a challenge, as the conclusions drawn by humans could not be applied to dogs due to many limitations. Therefore, two classical music pieces were chosen with particular characteristics of rhythm (andante, lento sostenuto), one of which (Mozart Κ.448) had already displayed positive effects both in humans [4,9,10,11] and rats [15,16,17], but had never been applied to dogs before the present study. Furthermore, the sound level was adjusted to 50 dB and never exceeded 65 dB because this is in accordance with the sound levels used for a dog’s auditory stimulation [20,21,23,24,25,26,35]. Although previous studies in animals have employed different music genres, we preferred to use only classical music pieces which met the previous standards, i.e., a slow tempo (andante, lento sostenuto), preset sound level (<80 dB), etc.

### 4.1. Sedation Scores

Significantly higher sedation scores were observed in the CM and ΜΜ treatment groups compared to the NM treatment group. As expected, the chosen pre-anaesthetic protocol induced mild sedation in the majority of dogs in all treatment groups, but exposure to either Chopin or Mozart music seemed to potentiate the level of sedation achieved. In humans, exposure to music during premedication with midazolam contributed to deeper sedation compared to patients who only received midazolam [1,38], while preoperative relaxing music of the patient’s choice reduced stress levels compared to the administration of midazolam [3]. Furthermore, 10 critically ill patients requiring intubation and ventilation achieved significantly deeper sedation when exposed to the slow movements of Mozart’s piano sonatas during their stay in the ICU compared to the controls [4]. In contrast to these aforementioned findings in humans, a study reported that the application of music in dogs premedicated with dexmedetomidine did not result in deeper sedation, as restraint test scores were not significantly different between the groups exposed to a background noise of 40–45 dB or music [26]. However, in that study, the music group was only compared to groups exposed to background noise (40–45 dB) or recorded human voices (55–60 dB), using sound levels similar to the music exposure, and not to a group of dogs who remained undisturbed and under total silence. In addition, another study reported that un-sedated dogs, when exposed to classical music, spent more time resting and less time barking, compared to dogs exposed to human conversation and other types of auditory stimulus; however, the authors do not mention the exact sound levels of the aforementioned acoustic stimuli [18]. Carrying on with the study of Albright et al. [26], it should be noted that dexmedetomidine is a potent sedative, especially when administered in rather high doses such as 10 μg/kg. Therefore, the potential impact of music on the level of sedation could be difficult to distinguish when using dexmedetomidine. This argument can be supported by the fact that under dexmedetomidine sedation, dogs in all groups did not display significant differences in their levels of sedation, even when exposed to a pretty loud environment consisting of 80–85 dB recorded human voices [26]. Perhaps the use of such a potent sedative affected these results. The choice of the premedication protocol in the present study aimed to avoid such an effect, given that dogs that received dexmedetomidine (10 μg/kg) were significantly more sedated than those that received acepromazine (0.05 mg/kg) [39].

### 4.2. Propofol Dose

The higher sedation scores observed in the music treatment groups (CM, MM) were followed by significantly lower propofol dose requirements compared to the NM treatment group. Therefore, dogs’ exposure to music (Chopin, Mozart) during the preoperative period seemed to lower significantly the propofol dose required for tracheal intubation by 18% and 26%, respectively. In humans [40], similar results were observed in a study in which the effect of exposure to music on propofol requirements was investigated in women undergoing breast surgery. In that study, the administration of propofol was titrated so that all patients achieved a similar anaesthetic depth, as indicated by a Bispectral Index (BIS) value of 70. The mean concentration of the propofol required was lower for the music group, but the difference was not statistically significant [40]. A positive effect of music has also been observed in a variety of surgical procedures, where groups exposed to music required significantly less propofol for the completion of each operation [2,6]. Finally, one more study evaluated the effect of preoperative music on the dose of propofol required for the induction of anaesthesia in humans, aiming at a BIS value of 60, without detecting any benefit for the music group [38]. To the authors’ knowledge, no studies evaluating the effect of music on propofol requirements for tracheal intubation have been conducted in domestic animals.

However, the propofol doses required for intubation in the present study were higher compared to the doses required for intubation in other studies with similar premedication protocols. In two studies in which dogs were premedicated with ACP 0.05 mg/kg and butorphanol 0.2 mg/kg, it was reported that either 3.8 ± 0.6 mg/kg or 3.6 ± 0.7 mg/kg propofol was required for the induction of anaesthesia [41,42] while the authors of one of these studies [42] declared that the anticipated propofol dose requirements were 3 to 6 mg/kg for the specific premedication protocol. These differences between those studies and ours may be due to the slightly higher dose of both the ACP and the butorphanol that were used in them. Furthermore, referring to the literature, when ACP was combined with buprenorphine (0.02 mg/kg) either intramuscularly or subcutaneously, the propofol dose for the intubation of the trachea was 2.4 ± 1.25 and 2.28 ± 0.843 mg/kg, respectively [36]. Our study utilized the same sedation scale and the same criteria for the assessment of the tracheal intubation conditions; however, the propofol doses used in our study seemed to be about two-fold higher compared to this particular [36] study. This difference may be due to the potentially distinct sedative effects of buprenorphine compared to butorphanol. It has been reported in the literature that the administration of μ-opioids results in mild sedation, whereas κ agonists produce only minimal sedation, as well [43]. Finally, when ACP 0.02–0.05 mg/kg was combined with morphine at 0.3–0.5 mg/kg, much higher propofol induction doses were reported, i.e., 4.6 to 6 mg/kg [44,45]. According to the required doses of propofol in these studies, someone might conclude that music does not lower induction requirements. However, we already know that music applied as a nonpharmacological approach exhibits only mild effects, and if we want to arrive at a safe conclusion about propofol while sparing the effects of music, we should compare mild premedication protocols while administering propofol in the same way (infusion allowing proper equilibration period between plasma and effect site).

### 4.3. Limitations of the Study

The fact that the dogs included in the present study were purpose-bred laboratory Beagles with a rather calm temperament and were familiarized with the staff and handling manipulations could be considered a potential limitation of this study. Perhaps if dogs of breeds with a more nervous temperament were used, the results would be different. Furthermore, another limitation could be the fact that we decided to use dogs that were unfamiliar with music listening as part of their daily routine: something that may have influenced our results.

Finally, the sedation scoring scale used in our study, i.e., the one proposed by Gurney et al. (2009) [36], was not validated. We recognize that this is not ideal, but during the time period our study was performed, Wagner et al. (2017) [46] had not published their validated scoring system. However, the scoring system of Gurney et al. (2009) [36] has been used plenty of times in published studies as the only sedation scoring system.

## 5. Conclusions

In conclusion, the results of the present study indicate that the exposure of dogs to classical music during the preoperative period has a beneficial effect on sedation and contributes to approximately 20% lower propofol dose requirements for the intubation of the trachea. Perhaps it would be of interest to evaluate the potential benefit of music applications not only during the preoperative period but also during the postoperative or even perioperative period in dogs, considering the advantages of music as a complementary tool during anaesthesia.

## Figures and Tables

**Table 1:** Subjective sedation scale [36] for assessment of sedation in 20 dogs exposed to Chopin (CM), Mozart (MM) and No music (NM) 45 min after premedication with ACP 0.03 mg/kg and butorphanol 0.1 mg/kg IM. Overall score 0–15 (0 = no sedation, 15 = well sedated).

Composite Descriptive Sedation Score	
**Spontaneous posture**	
standing	0
sternally recumbent	1
laterally recumbent	2
**Palpebral reflex**	
brisk	0
slow	1
absent	2
**Eye position**	
forward (normal position)	0
rotated ventrally	2
**Response to sound (handclap)**	
Body movement	0
Head movement	1
Ear twitch	2
No reaction	3
**Resistance to lateral recumbency**	
full (stands)	0
moderate restraint required	1
mild restraint required	2
no resistance	3
**Overall appearance**	
no sedation apparent	0
mild sedation	1
moderate sedation	2
well sedated	3

**Table 2:** Minimum, median, maximum, 25th percentile, 75th percentile values and interquartile range (IQR) of the sedation score in 20 dogs exposed to Chopin (CM), Mozart (MM) and No music (NM) for 90 min in total and 45 min after premedication.

	Treatment	Min	25th	Median	75th	Max	IQR	K-W ^1^ (Sig ^2^)	Post Hoc
Sedation Score	Chopin (CM)	1	3.5	5.0	6.0	8.0	2.5	20.031 (<0.01)	CM > NM MM > NM
	Mozart (MM)	1	4.0	6.0	8.0	10.0	4.0		
	No music (NM)	1	2.0	2.0	3.5	6.0	1.5		

^1^ K-W, Kruskal–Wallis test; ^2^ sig, level of significance.

**Table 3:** Minimum, mean, standard deviation (SD) and maximum propofol dose (mg/kg) in 20 dogs exposed to CM, MM and NM for 90 min during the preoperative period.

	Treatment	Min	Mean	SD	Max	F ^1^ (Sig ^2^)	Post Hoc
Propofol dose (mg/kg)	Chopin (CM)	3.0	4.7	0.9	6.0	16.508 (<0.01)	CM < NM MM < NM
	Mozart (MM)	3.0	4.2	0.8	5.5		
	No music (NM)	4.0	5.7	0.9	7.0		

^1^ F, F value; ^2^ sig, level of significance.

## Data Availability

Not applicable.
